# Surveillance of people with previously successfully treated diabetic macular oedema and proliferative diabetic retinopathy by trained ophthalmic graders: cost analysis from the EMERALD study

**DOI:** 10.1136/bjophthalmol-2021-318816

**Published:** 2021-06-03

**Authors:** Mandy Maredza, Hema Mistry, Noemi Lois, Steve Aldington, Norman Waugh, EMERALD Study Group, Ahmed Saad

**Affiliations:** 1 Warwick Medical School, University of Warwick, Coventry, UK; 2 The Wellcome-Wolfson Institute for Experimental Medicine, Queens University Belfast, Belfast, UK; 3 Gloucestershire Hospitals NHS Foundation Trust, Gloucester, UK

**Keywords:** imaging, macula, retina, diagnostic tests/investigation, public health

## Abstract

**Background/aims:**

Surveillance of people with previously successfully treated diabetic macular oedema (DMO) and proliferative diabetic retinopathy (PDR) adds pressure on ophthalmology services. This study evaluated a new surveillance pathway entailing multimodal imaging reviewed by trained ophthalmic graders and compared it with the current standard care (face-to-face evaluation by an ophthalmologist).

**Methods:**

Cost analysis of the new ophthalmic grader pathway, compared with the standard of care, from the perspective of the UK National Health Service, based on evidence from the Effectiveness of Multimodal imaging for the Evaluation of Retinal oedema And new vesseLs in Diabetic retinopathy study. Resource use data were prospectively obtained including times to undertake each procedure. Effectiveness was assessed in terms of sensitivity and specificity of referral decisions in the grader pathway. Costs (SDs) were analysed per 100 patients separately for DMO and PDR at 2018/2019 costs.

**Results:**

For DMO, where sensitivity was very high (97%), the cost difference (savings) for the grader’s pathway would be £1390 per 100 patients. For PDR, the cost would be reduced by £461 for seven-field Early Treatment for Diabetic Retinopathy Study (ETDRS) images and by £1889 for ultrawide field images, per 100 patients. Ultrawide images required less time to be obtained and read than seven-field ETDRS. The real savings would be in ophthalmologist time, which could be then redirected to the evaluation of people at high risk of visual loss.

**Conclusions:**

Surveillance of people with previously successfully treated DMO and PDR by trained ophthalmic graders can achieve satisfactory results and release ophthalmologist time.

**Trial registration numbers:**

NCT03490318, ISRCTN10856638.

## Introduction

Retinal complications of diabetes, namely diabetic macular oedema (DMO) and proliferative diabetic retinopathy (PDR), can lead to sight loss. In many people, DMO and PDR are treated successfully with laser photocoagulation and/or antivascular endothelial growth factor (anti-VEGF) therapies. However, both can recur following successful treatment, so long-term surveillance is necessary.

The number of people with diabetes is increasing and the numbers attending ophthalmology clinics continue to rise, despite improvements in glycaemic control and screening for DR have reduced the risk of advanced retinopathy.^1^ This makes it difficult for ophthalmology clinics to meet the demand and, as a result, detrimental delays in the evaluation and treatment of patients occur.^2^

National Institute for Health and Care Excellence (NICE) recommends treatment of severe DMO (with central retinal thickness on spectral-domain optical coherence tomography (SD-OCT) of >400 µm) with anti-VEGFs after which patients should usually be followed monthly during the first year of treatment, and every 1–3 months thereafter to detect recurrence.^3 4^ In less severe cases, NICE advices macular laser, which is applied in a single session and patients followed subsequently every 3–4 months.

PDR is currently treated with laser panretinal photocoagulation (PRP); treatment with anti-VEGF drugs is being introduced.^5^ After PRP, patients are followed at 6–12 monthly intervals to detect recurrence.

Given the chronic nature of DMO and PDR and the possibility of recurrence, follow-up is lifelong.

Currently in the National Health Service (NHS), ophthalmologists assess patients during follow-up using SD-OCT for DMO and fundus examination by slit-lamp biomicroscopy for PDR. Retinal photographs are not routinely taken to evaluate PDR.

Standard cameras cannot image the retinal periphery and much of the retina is not photographed in the standard seven-field Early Treatment for Diabetic Retinopathy Study (ETDRS) images. Newer ultrawide field (UWF) imaging (Optos, Dunfermline, Scotland, UK) captures nearly the entire retina in a single image; with three images (centre, superior and inferior) the whole retina is covered.

The Effectiveness of Multimodal imaging for the Evaluation of Retinal oedema And new vesseLs in Diabetic retinopathy (EMERALD) study assessed the diagnostic accuracy of a new surveillance pathway (ophthalmic grader pathway) compared with the current standard of care (ophthalmologist face-to-face examination) for people with previously successfully treated DMO and/or PDR. Diagnostic accuracy results of EMERALD have been reported.^6^ Herein, we present cost analyses, comparing the ophthalmic grader and standard of care pathways. If the grader pathway is as sensitive at detecting reactivation, graders could take over much of the routine surveillance. Specificity is also important, since the proportion referred by graders to ophthalmologists determines the savings in their time. We also compare costs of UWF fundus images with those of seven-field ETDRS.

## Patients and methods

### Study background

EMERALD was a diagnostic accuracy study^7^ in adults with type 1 or 2 diabetes and previously successfully treated DMO/PDR who, at the time of enrolment, had reactivated or inactive disease, recruited from 13 ophthalmology departments in the UK. The primary outcome was sensitivity of the ophthalmic grader pathway to detect presence of DMO and active PDR, using standard care as reference standard, which included slit-lamp examination and review of SD-OCT scans by an ophthalmologist.

The ophthalmic grader pathway entailed review of SD-OCT scans to detect DMO, and seven-field ETDRS and UWF images to detect PDR, by trained, tested and certified ophthalmic graders. The analysis took into consideration ophthalmic graders referring to ophthalmologists all patients diagnosed as having DMO and/or active PDR, or if they were uncertain about the diagnosis, or if images were ungradable, as this is what would occur in clinical practice if the pathway is implemented. As new vessels may be seen on fundus images but missed on fundus examination, the study included an enhanced reference standard for PDR. The latter combined results of face to face examination by the ophthalmologists with the evaluation of seven-field ETDRS and UWF images by an ophthalmologist expert in DR; if any detected active PDR, the grading, based on the enhanced reference standard, was active PDR.

### Cost analysis

Costs of standard care, ophthalmic grader pathway and enhanced reference standard included staff costs (based on time undertaken to perform activities/procedures), equipment costs and costs of training graders. The cost analysis was undertaken from the perspective of the UK NHS.^3^ All costs were at a single time point, expressed in £ sterling and valued in 2018–2019 prices.

### Measurement and valuation of resource use

Resource use was captured prospectively on case report forms (CRFs) at each participant’s routine standard care visit. CRFs captured the time taken to:

- Undertake best-corrected visual acuity.
- Obtain SD-OCT, seven-field ETDRS and UWF images by ophthalmic photographers/imaging technicians.
- Undertake face-to-face examination by the ophthalmologist, including slit-lamp biomicroscopy, review of SD-OCT scans (to detect DMO) and counselling the patient. However, for the base-case analysis, we used the NHS standard reference cost for an Ophthalmology outpatient visit to cost the standard care pathway. That unit cost captures the reading of SD-OCT by ophthalmologists.
- Grade SD-OCT, seven-field ETDRS and UWF images by ophthalmic graders.
- Grade seven-field and UWF images by the ophthalmologist to set the enhanced reference standard.

Times were converted into staff costs by multiplying the number of hours that staff worked by their hourly salary. We obtained hourly staff costs from the Personal Social Services Research Unit's (PSSRU’s) Unit Costs of Health and Social Care compendium^8^ and NHS reference costs.^9^ Graders were costed at band 7 salary scale while photographers/imaging technicians at band 6 level (online supplemental appendix, table A1). All staff costs reflect NHS salary, superannuation, national insurance and overhead costs.

10.1136/bjophthalmol-2021-318816.supp1 Supplementary data

To calculate an annual equivalent cost of equipment, we annuitised capital costs of the item over its useful life span, applying a discount rate of 3.5% per annum (online supplemental appendix, table A2).^10^ Equipment cost (including annual maintenance costs) was used to derive a per-patient cost of equipment assuming that outpatient throughput for the eye department is 9000 patient visits per year.

Costs of training graders were based on the training delivered in EMERALD to ophthalmic graders and included: (1) costs of preparing training materials; (2) time costs of two trainers delivering a face-to-face meeting over 2 days; (3) salary costs of graders for 2 days; (4) costs of travel to attend the face-to-face meeting; (5) costs of half-day web-based ‘further’ training for all graders, (6) costs of half-day web-based training for graders who failed the test at first attempt (who had the opportunity to retake it again) and (7) costs of live webinar licence. To follow recommendation that investment costs of education should always be included when evaluating the cost-effectiveness of different approaches to using health service staff^11^ we discounted training costs at 3.5% p.a., assuming effects of training received will last approximately 10 years. Further details on how graders were trained are reported elsewhere.^6^

### Sensitivity analyses

We compared costs of delivering the pathways based on the main analysis but also based on other scenarios (eg, specifically detecting active disease), as prespecified in the EMERALD Study Statistical Analysis Plan (online supplemental appendix, table A3). In addition, we conducted an exploratory evaluation to assess graders’ performance in referring active PDR against an enhanced reference standard consisting of the ophthalmologist face-to-face examination supplemented by reading of retinal photographs. For costing purposes, costs of the enhanced reference standard included reading only one set of images, as this is how it would be done if the pathway were to be implemented.

### Analyses of cost data

We estimated mean costs and SDs of each procedure, including obtaining and reading images (UWF, OCT and seven-field ETDRS). The costs (SDs) are presented per 100 patients. For the standard care pathway costs are simply the reference standard costs for an ophthalmology outpatient visit multiplied by 100, which include taking and reading SD-OCT images. Costs for the grader pathway options take into account the specificity. Thus, if the specificity of the grader pathway was 50%, half the patients with no active disease would be seen by ophthalmologists. The final costs for the grader pathway would therefore include the costs of photographer taking images+grader reading images for 100 patients+ophthalmologists’ time of reviewing patients classified as possibly active DMO/PDR when DMO/PDR is actually inactive, based on specificity.

### Missing data

EMERALD was a cross-sectional study with no follow-up. Images were taken on the same day patients saw the ophthalmologist and CRFs were completed then. Thus, lost to follow-up was not an issue. In a few individuals recorded values seemed unrealistic, for example, where times to perform a task were close to 0 or more than 60 min and when images were missing. We excluded these from the analysis.

### Longer-term economic modelling

The study protocol allowed for decision-analytical modelling to estimate the longer-term cost-effectiveness of the ophthalmic grader pathway. Use of a lifelong time horizon may be warranted if observed differences in sensitivity between the new pathway tested and the current standard care were sufficient to affect visual outcomes for either PDR or DMO. We concluded that long-term modelling was not necessary, as sensitivity of the new pathway was similar to that of current standard care for DMO. For PDR, the overall sensitivity was lower but considered acceptable, especially for high-risk PDR. In addition, there are data gaps for several key items, including rate of progression and visual consequences of re-activated PDR in people previously treated with PRP, and the proportion that would be picked up at the next routine visit.

## Results

Table 1 shows the average and median time taken in minutes to complete specified procedures by ophthalmologists, graders and imaging technicians.

**Table 1:** Time (in minutes) taken to complete specified activities per patient by ophthalmic photographers/imaging technicians, ophthalmic graders and ophthalmologists

Time-related activity	N	Mean	SD	Median	Q1	Q3	IQR
Standard care**	380	15.3	7.6	14.0	10.0	19.0	9.0
Ophthalmic photographer/imaging technician obtaining seven-field ETDRS images	267	15.1	8.4	13.0	10.0	18.0	8.0
Ophthalmic photographer/imaging technician obtaining UWF images	272	10.3	6.6	9.0	5.0	14.0	9.0
Ophthalmic grader reading seven-field ETDRS images	267	10.6	6.5	9.0	7.0	13.0	6.0
Ophthalmic grader reading UWF images	264	9.3	5.9	8.0	5.0	11.5	6.5
Ophthalmologist reading seven-field ETDRS images	268	11.9	5.00	11.0	9.0	15.0	6
Ophthalmologists reading UWF images	268	10.1	4.0	9.5	7.0	12.0	5.0
Ophthalmic grader reading SD-OCT images	248	4.2	3.3	4.0	3.0	5.0	2.0

The standard of care pathway includes all patients (n=380 of the full total of 397 in EMERALD; 17 are not included as the times provided for some of the procedures were considered not plausible, being either too high or too low). Note there were 272 patients eligible for grader pathway with DMO and 281 eligible with PDR.

*Ophthalmologists face-to-face evaluation of patients with access to SD-OCT images.

EMERALD, Effectiveness of Multimodal imaging for the Evaluation of Retinal oedema And new vesseLs in Diabetic retinopathy; ETDRS, Early treatment diabetic retinopathy study; Q1, Quartile 1; Q3, Quartile 3; SD-OCT, spectral domain optical coherence tomography; UWF, ultra wide field.

Table 2 presents costs of procedures. Costs for obtaining and reading images were lower with UWF compared with seven-field ETDRS. We estimated an annual discounted cost of training graders of £198.70 per grader, but this cost is trivial once divided by the number of patient images that graders would read in a year.

**Table 2:** Costs of procedures

Item	Cost per patient (SD)	Notes
Standard care* (base case)	£58.00	NHS 2019/2020 national tariff for an ophthalmology outpatient follow-up appointment with a consultant
Ophthalmic photographer/imaging technician obtaining seven-field ETDRS images (band 6)	£12.78 (6.83)	Time cost × photographer salary+equipment cost
Ophthalmic photographer/imaging technician obtaining UWF images (Band 6)	£9.75 (5.85)	Time cost × photographer salary+equipment cost
Reading ETDRS images by ophthalmic grader (Band 7)	£10.45 (6.40)	Time cost × grader salary
Reading UWF images by ophthalmic grader (Band 7)	£9.10 (5.77)	Time cost × grader salary
Reading ETDRS images by ophthalmologist	£21.58 (9.02)	Time cost × salary
Reading UWF images by ophthalmologist	£18.23 (7.28)	Time cost × salary
Taking SD-OCT	Occurs in both pathways	Cost not included
Reading SD-OCT—grader (band 7)	£4.08 (3.25)	Time cost × salary

Equipment cost is a small % of total cost (see online supplemental appendix table A2). Salary costs include employers’ costs at 20%. Reading SD-OCT by ophthalmologist is included in the NHS reference cost for the standard care pathway.

*Ophthalmologists face to face evaluation of patients with access to SD-OCT images.

ETDRS, Early Treatment Diabetic Retinopathy Study; NHS, National Health Service; SD-OCT, spectral domain optical coherence tomography; UWF, ultrawide field.

### Costs of surveillance of people with DMO and PDR

#### DMO: comparison of ophthalmic grader pathway with current standard care

Table 3 shows costs of follow-up of DMO by ophthalmic graders and ophthalmologists. The cost analysis takes into account referrals by the grader, which would include those related to ‘active DMO’ (ie, grader believes there is recurrence of DMO), ‘unsure’ (ie, grader thinks there may be recurrence of DMO but is not fully sure) and ‘ungradable’ (ie, reader is unable to grade the image). The estimated mean cost of surveillance of people with DMO per 100 patients was £4410.00 for ophthalmic graders compared with £5800.00 for standard care. The mean difference (cost savings) per 100 patients would be £1390.00. The real savings would be in ophthalmologist time, released for other purposes. It should be noted that savings would only apply if neither eye had DMO nor active PDR as otherwise the patient would still require an ophthalmologist evaluation.

**Table 3:** Relative costs of surveillance of people with previously successfully treated DMO

	Sensitivity	Specificity	Cost per 100 patients
Standard care pathway *	Assumed 100%	Assumed 100%	£5800.00†
Grader pathway (band 7)	97%	31%	(4.08×100) +(69‡ x 58) = £4410.00§
Cost difference			£1390.00¶

*Standard care pathway=ophthalmologists face-to-face evaluation of patients with access to SD-OCT images.

†Costs for 100 patients in the standard of care pathway.

‡Ophthalmologists' time of reviewing 69% of patients classified as possibly active DMO when DMO is inactive, based on specificity.

§Ophthalmic grader costs for reading 100 OCTs+Ophthalmologist costs for 69 x standard care visits.

¶Current standard-of-care costs minus ophthalmic grader costs.

DMO, diabetic macular oedema; SD-OCT, spectral-domain optical coherence tomography.

All sensitivity analyses (SENA) (online supplemental appendix, tables A4–A7) supported the main finding that surveillance by graders could provide useful savings in ophthalmologist time. Cost savings per 100 patients could range between £810.00 (if graders were asked to identify any active DMO in either eye possibly requiring treatment) to £2840.00 (if graders were asked to identify only central involving active DMO), because of differences in specificity affecting the proportion of people referred to ophthalmologists. Specificity is the main factor driving cost differences.

#### PDR: comparison of ophthalmic grader pathway with current standard care

Table 4 shows costs of surveillance of PDR comparing ophthalmic grader’s pathway with the standard care. Cost savings from graders reading the seven-field ETDRS and UWF images could be £461.00 and £1889.00 per 100 follow-up visits respectively, because the UWF images take less time to read. The time to obtain and read images (table 1) is the main driver of the differences of costs since the equipment costs were small.

**Table 4:** Cost comparison—ophthalmic grader pathway and standard care pathway

	Sensitivity	Specificity	Cost per 100 patients	Cost saving compared with standard care*
Standard care pathway ††	Assumed 100%	Assumed 100%	£5800.00	–
Ophthalmic grader evaluating seven-field ETDRS images*	85%	48%	£5339.00	£461.00‡
Ophthalmic grader evaluating UWF images*	83%	54%	£4611.00	£1189.00§

*The analysis from which the sensitivity and specificity values are derived in this table are based on either eye being active and referrals to ophthalmologist. *=Grader referral for PDR = ‘active’ + ‘unsure’ + ‘ungradable’. The cost will not be increased if both eyes show active PDR.

†Standard care pathway=ophthalmologist face-to-face examination for active PDR in either eye.

‡Calculated as (time of taking ETDRS photos+costs of ophthalmic grader reading ETDRS photos) x100 +(52 × standard of care costs). Because of the specificity, 52% of patients will be referred to the ophthalmologist.

§Calculated as (time of taking UWF fundus images+costs of ophthalmic grader reading UWF images)*100 +(46 * standard of care costs). Because of the specificity, 46% of patients will be referred to the ophthalmologist.

ETDRS, Early Treatment for Diabetic Retinopathy Study; PDR, proliferative diabetic retinopath; UWF, ultrawide field.

Most (SENA 1 and SENA 2: online supplemental appendix, tables A8–9) corroborate the main finding that the ophthalmic grader pathway could save ophthalmologist time. Savings would be modest or even zero in SENA 4 (online supplemental table A10) for ophthalmic graders evaluating seven-field ETDRS images for people with pre-retinal or vitreous haemorrhages (ie, high-risk PDR); in this group there would be savings with UWF imaging.

#### PDR; comparison with enhanced reference standard

In EMERALD, both ETRDS and UWF images were examined in the enhanced standard. If the standard of care for PDR were to be supplemented with images in clinical practice, only one set of images would be used. Based on the results, we have assumed these would be UWF images.

Online supplemental appendix, table A11 shows an analysis comparing the ophthalmic graders referral for either eye by UWF imaging against standard care combined with either UWF imaging or seven-field ETDRS as graded by the ophthalmologists of active PDR in either eye. Costs using UWF imaging are lower than those using seven-field ETDRS images. So, when comparing enhanced standard care with the grader pathway, costs of UWF images are used. The grader pathway is about half the cost of the enhanced reference standard (table 5).

**Table 5:** Cost and consequence comparison of enhanced reference standard* and grader pathway

	Sensitivity	Specificity	Cost per 100 patients	Cost savings
Standard of care+UWF images*	Assumed 100%	Assumed 100%	£8598	–
Ophthalmic Grader evaluating UWF images†	79.7%	58.8%	£4263	£4335

*Ophthalmologists face-to-face evaluation of patients with access to SD-OCT images supplemented by the reading by the ophthalmologists of the UWF images.

†In the main analysis, ophthalmic graders refer to ophthalmologists all patients identified as having ‘active PDR’, but also if they were ‘uncertain’ about the diagnosis of if ‘ungradable’ images.

UWF, ultrawide field.

## Discussion

Follow-up of DMO after treatment by trained and tested ophthalmic graders using SD-OCT was found to have very good sensitivity (97%), but lower specificity, ranging from 31% to 56% depending on the scenario investigated. Even that level of specificity would allow useful savings in ophthalmologist time, which could be directed for example, to the care of other people with visual threatening disease. In PDR, the overall sensitivity of the ophthalmic grader pathway was lower than that of DMO, but the sensitivity was higher in high risk PDR (pre-retinal and/or vitreous haemorrhage), nearly reaching 90% if UWF imaging was used.

In EMERALD, images were evaluated without any information about the patient, with ophthalmic graders (and ophthalmologists undertaking the enhanced reference standard) masked to clinical data and previous images. Specificity is an important factor in the costs of the grader pathway. In routine care it is likely that having access to clinical information and previous images would improve sensitivity and specificity of the grader pathway. For example, it is possible that in the medical records the clinician would have recorded there was a macular cyst on SD-OCT at the time treatment was stopped; in that case it is likely the grader would not refer the patient. Previous images would enable for graders to check whether any lesion seen, for example, new vessels elsewhere, had been there previously, and if so, whether they were unchanged or improved/resolved, or whether they were a new finding, reducing inappropriate referrals and improving specificity. Access to medical records and previous images is now facilitated by the use of electronic medical records across the UK (eg, Medisoft, MediSIGHT, OpenEyes) and already existing and new platforms (eg, Ophthalsuite) to view fundus images and SD-OCTs.

One option would be that if the grader sees a questionable feature, annotates it (to save ophthalmologist time) and submits the image(s) (not the patient) for review by the ophthalmologist. This would avoid a proportion of potential referrals.

A more efficient option, although more difficult to organise, would be a ‘one-stop’ system in which patients are imaged, images graded on the same day (an ophthalmologist could be available for advice in situ or remotely), and patient informed of the results immediately. As found in the qualitative work undertaken in EMERALD (see accompanying manuscript by Prior and Lois), this would be preferred by patients.

Given the very high number of people referred to ophthalmology clinics with DMO and PDR, clinics are finding it difficult to cope with the demand, resulting in delays in evaluation and treatment of patients.^12^ Delayed treatment leads not only to sight loss,^2^ but can reduce the cost-effectiveness of therapies. For example, a reduction in the number of anti-VEGF injections during the first year of treatment if patients are not seen and treated promptly, would lead to a lesser visual acuity improvement.^13^ Furthermore, as a result of these increased waiting times, the NHS often subcontracts work to private clinics or employs locum staff to cope with the demand; an expensive exercise. EMERALD showed the new pathway could save ophthalmologist time; releasing ophthalmologists to see or treat other patients could reduce the need for frequent NHS waiting list initiatives, saving money to the NHS. Whether or not, however, the ophthalmologist’s time released is used efficiently in the NHS for other purposes and whether savings are realised will depend on an efficient reorganisation of services.

We used national reference costs for England of an ophthalmology outpatient appointment. These have sometimes been criticised for underestimating true costs, including at NICE Technology Appraisal Committee meetings, as noted in the NICE guidance on ranibizumab for DMO (TA 274). The true cost of an outpatient visit, however, is not known.

Times required to obtain and evaluate UWF images were shorter than those of seven-field ETDRS images with comparable sensitivity (higher for high risk PDR). This implies that if imaging is added, UWF should be used. In EMERALD, UWF images included three fields; if one were to provide comparable sensitivity and specificity, this would reduce time to obtain and grade images. In EMERALD images were obtained following pupillary dilation. If a single UWF image obtained without mydriasis provided comparable sensitivity and specificity, patient satisfaction would probably increase. Patients would be able to drive following the examination, and time in clinic would be reduced, as they would not need to wait for their pupils to dilate prior to imaging. It would also lead to some savings from dilating drops not being required.

We provided training costs for graders as observed in EMERALD. If ophthalmic grading is introduced in practice, other arrangements might be made, including distant online training. Once a comprehensive training set of images of previously treated patients had been developed, graders could learn online at their own pace. This would remove travel costs to face to face meetings, which are less appropriate in the COVID-19 era. There would be initial costs of creating sets of training images and supporting material, but there would be no costs of ophthalmologist or other trainers doing face-to-face teaching. There would be a need for a regular quality assurance system after initial training.

EMERALD did not assess the marginal benefits of adding the reading of UWF images by ophthalmologists to standard care. The marginal cost of this would be ~£28 per patient.

If sensitivity for detecting reactivated PDR was lower than ideal and some people with active PDR were missed, the consequences would depend on the rate of progression of the disease. This would be expected to be slower in patients previously treated by PRP, as those included in EMERALD, than in those with treatment-naïve PDR. Furthermore, reactivation might be identified at the next follow-up visit before any harm resulted. Moreover, patients themselves would be likely to seek care if they were to experience floaters or sight loss, for example, due to a vitreous haemorrhage. It is possible that in the future some people with PDR will be treated with anti-VEGFs, and the rate of progression of reactivated PDR after anti-VEGF treatment is unknown. It might be faster than after PRP and the consequences more severe.^14 15^

In conclusion, EMERALD showed that surveillance of people with previously successfully treated DMO and PDR by ophthalmic graders using multimodal imaging can reduce costs and release ophthalmologist time.

## Data Availability

Data are available on reasonable request. The authors would consider sharing of data result of this work upon reasonable request and following approval by the sponsor and the EMERALD Study Group.
