# Supplementary material for: Surveillance of people with previously successfully treated diabetic macular oedema and proliferative diabetic retinopathy by trained ophthalmic graders: cost analysis from the EMERALD study
Source: Br J Ophthalmol. 2021 Jun 3;106(11):1549–54. doi: 10.1136/bjophthalmol-2021-318816 (PMC9606536; doi:10.1136/bjophthalmol-2021-318816)
Supplement: Supplementary data [file bjophthalmol-2021-318816supp001.pdf]

Supplementary Appendix to EMERALD cost analysis paper

Supplementary Table A1 : Staff costs (2019) prices

Resource Item	Afc * Band	Unit Cost	Measurement unit	Source
Ophthalmic photographer/ Imaging technician	6	£49.00	per working hr	PSSRU 2019, page 143[4] †
Ophthalmic grader	7	£59.00	per working hour	PSSRU 2019, page 143 [4]
Ophthalmologist (consultant medical)	N/A	£109.00	per working hour	PSSRU 2019, page 150 [4]
Associate specialist	N/A	£108.00	per working hour	PSSRU 2019, page 150 [4]
Specialty Registrar ‡	N/A	£47.00	per working hour	PSSRU 2019, page 150 [4]
Ophthalmologist (average cost)	N/A	£108.50	per working hour	Calculated §
Ophthalmologist outpatient follow-up appointment (slit-lamp examination)	N/A	£58.00	per patient contact	NHS 2019/20 national tariff payment system [5]

Supplementary Table A2: Unit costs for equipment (2019) prices

Cost variable	Current Cost	Lifespan	Annual throughput **	Total annual discounted costs	Cost per patient
7-field ETDRS imaging camera ***Topcon TRC-MW-8***	£14,500 - purchase price<br><br>£500 – annual maintenance costs costed from year 3 to year 8 ††	8	9,000	£2,431.18	£0.27
Ultra-wide angle imaging equipment ***Optos California aqua RG/AF/FA/ICG***	£88,255 - purchase price ‡‡<br>£5,250 - Maintenance cost sold as extended warranty (once-off fee)	10	9,000	£11,243.17	£1.25
Slit lamp ***Haag-Streit-BM-900-Table-LED-Slit-Lamp***	£11,300 -Purchase price §§c<br>£1,750 - Maintenance cost sold as extended warranty (once-off fee)	10	9,000	£1,569.15	£0.17

Supplementary Table A3: List of sensitivity analyses of diagnostic accuracy

**Analysis name**	**Level of Analysis**	**DMO index test positive**	**DMO reference standard**	**PDR index test positive**	**PDR reference standard**
SENA1	Person	OCT based ophthalmic grader **identification of active disease** in either eye	O-FTF+OCT assessment of active DMO in either eye	**OPTOS based** ophthalmic grader **identification of active disease** in either eye/ **ETDRS based** ophthalmic grader identification of **active disease** in either eye	O-FTF assessment of active PDR in either eye
SENA2	Person	OCT based ophthalmic grader **referral***** for either eye	O-FTF+OCT assessment of active DMO in either eye **requiring treatment**	**OPTOS based** ophthalmic grader referral for either eye/ **ETDRS based** ophthalmic grader referral for either eye	O-FTF assessment of active PDR in either eye **requiring treatment**
SENA3	Person	OCT based ophthalmic grader **identification of central involving DMO** in either eye	O-FTF+OCT assessment of central involving DMO in either eye	N/A	N/A
SENA4	Person	N/A	N/A	**OPTOS based** ophthalmic grader **referral** for either eye/ **ETDRS based** ophthalmic grader **referral** for either eye	O-FTF assessment of active **PDR with pre-retinal or vitreous haemorrhage** in either eye
SENA5	Person	N/A	N/A	**OPTOS based** ophthalmic grader referral for either eye/ **ETDRS based** ophthalmic grader **referral** for either eye	**Enhanced standard**
SENA6	Person	OCT based ophthalmic grader **referral** for either eye [**participants assessed in routine clinic** setting only]	O-FTF+OCT assessment of active DMO in either eye [**participants assessed in routine clinic setting only**]	**OPTOS based** ophthalmic grader referral for either eye [**participants assessed in routine clinic setting only]** / **ETDRS based** ophthalmic grader referral for either eye [**participants assessed in routine clinic** setting only]	O-FTF assessment of active PDR in either eye [participants assessed in routine clinic setting only]

[*] Agenda for change pay scale band
[†] We chose the cost per hourly rate of radiographers as the more relevant unit cost for ophthalmic imaging technicians

‡ Although patients may be seen by specialty registrars, we assumed that unless advanced in training, most will be closely supervised by associate specialists or consultants hence the average unit cost for an ophthalmologist (used in current analysis) is based on associate specialist and consultant salaries
§ Average unit cost for an ophthalmologist based on associate specialist and consultant salaries
N/A = Not applicable
** Annual throughput estimate: (Lois, N; personal communication, 6 January 2020)
†† Equipment price quotation (F Byron, *Topcon Ireland Medical*, Dublin, Ireland, personal communication, 10 December 2019)
‡‡ Equipment price quotation (A Brown, *Optos Plc*, Dunfermline, Scotland, personal communication, 9 December 2019)
§§ Equipment price: Veatch Ophthalmic Instruments
ETDRS = Early Treatment Diabetic Retinopathy Study
*** grader referral for DMO or PDR= "active" + "unsure" + "ungradable". N/A = not applicable. DMO = diabetic macular oedema; PDR = proliferative diabetic retinopathy; ETDRS = Early Treatment Diabetic Retinopathy Study

COST-COMPARISON OPHTHALMIC GRADER PATHWAY VS STANDARD OF CARE FOR DMO

Supplementary Table A4: Cost comparison of ophthalmic grader pathway and standard care for diabetic macular oedema (SENA 1)

Sensitivity Analysis (SENA) Scenario	Sensitivity	Specificity	Cost per 100 patients
**Active DMO in either eye based on the reference standard***	Assumed 100%	Assumed 100%	£5,800.00
**G-OCT identified active DMO**	95%	38%	£4,004.00
**Cost difference**			£1,796.00

* = ophthalmologist face-to-face examination with access to spectral domain optical coherence tomography images; G-OCT = Grader identification of active DMO using OCT images (unsure or ungradable not included); SENA = sensitivity analysis.

In SENA 1 (where the diagnostic performance of graders for active DMO [excluding uncertain and ungradable] was assessed against the reference standard) specificity improved.

Supplementary Table A5: Cost comparison of ophthalmic grader pathway and standard care for diabetic macular oedema (SENA 2)

Sensitivity Analysis (SENA) Scenario	Sensitivity	Specificity	Cost per 100 patients
**Active DMO in either eye requiring treatment based on the reference Standard***	Assumed 100%	Assumed 100%	£5,800.00
**G-OCT referral for DMO**	95%	21%	£4,990.00
**Cost difference**			£810.00

* = ophthalmologist face-to-face examination with access to spectral domain optical coherence tomography images; G-OCT referral for DMO = ophthalmic grader referral includes those cases graders considered "active DMO", "unsure" and "ungradable", based on spectral domain optical coherence tomography images.

Supplementary Table A6: Cost comparison of ophthalmic grader pathway and standard care for diabetic macular oedema (SENA 3)

Sensitivity Analysis (SENA) Scenario	Sensitivity	Specificity	Cost per 100 patients
**Central involving active DMO in either eye based on the reference standard***	Assumed 100%	Assumed 100%	£5,800.00
**G-OCT identified central involving DMO**	94%	56%	£2,960.00
**Cost difference**			£2,840.00

* = ophthalmologist face-to-face examination with access to spectral domain optical coherence tomography images; G-OCT = Grader assessment based on spectral domain optical coherence tomography images.

Supplementary Table A7: Cost comparison of ophthalmic grader pathway and standard care for diabetic macular oedema (SENA 6)

Sensitivity Analysis (SENA) Scenario	Sensitivity	Specificity	Cost per 100 patients
**Standard care identification of active DMO in either eye in routine clinic (rather than in research clinic)***	Assumed 100%	Assumed 100%	£5,800.00
**G-OCT referral for DMO in routine clinic**	95%	40%	£3,888.00
**Cost difference**			£1,912.00

* = ophthalmologist face-to-face examination with access to spectral domain optical coherence tomography images; G-OCT referral for DMO = ophthalmic grader referral includes those graders considered “active DMO”, “unsure” and “ungradable”, based on spectral domain optical coherence tomography images of patients assessed in routine clinics (rather than in research clinics).

COST-COMPARISON OPHTHALMIC GRADER PATHWAY VS STANDARD OF CARE FOR PDR

Supplementary Table A8: Cost comparison of ophthalmic grader pathway and standard care for proliferative diabetic retinopathy (SENA 1)

Sensitivity Analysis (SENA) Scenario	Sensitivity	Specificity	Cost per 100 patients	Cost saving compared to standard care
**Active PDR in either eye based on the reference standard***	Assumed 100%	Assumed 100%	£5,800.00	-
**G-ETDRS identified active PDR**	71.00%	70.00%	£4,063.00	£1,737.00
**G-OPTOS identified active PDR**	63.00%	73.00%	£3,451.00	£2,349.00

* = ophthalmologist face-to-face examination; G-ETDRS = ophthalmic grader identifying active PDR based on 7 field ETDRS images; G-OPTOS = ophthalmic grader identifying active PDR based on ultra-wide field Optos images. ETDRS = Early Treatment Diabetic Retinopathy.

The cost-difference (savings) for the ophthalmic grader pathway were higher in this scenario (£1,737 vs £2,349) for UWF and 7-field ETDRS images, respectively (Table A8), but sensitivity was too poor to be acceptable. However, in practice the grader pathway would not be implemented in this way because graders in routine care would be referring not only patients in whom they identified active disease but also those in whom they are unsure and those with ungradable images.

Supplementary Table A 9: Cost comparison of ophthalmic grader pathway and standard care for proliferative diabetic retinopathy (SENA 2)

Sensitivity Analysis (SENA) Scenario	Sensitivity	Specificity	Cost per 100 patients	Cost saving compared to standard care
**Active PDR in either eye based on the reference standard***	Assumed 100%	Assumed 100%	£5,800.00	-
**G-ETDRS referral for PDR**	88.00%	46.00%	£5,455.00	£345.00
**G-OPTOS referral for PDR**	86.00%	52.00%	£4,669.00	£1,131.00

* = ophthalmologist face-to-face examination; G-ETDRS referral for PDR = referral for PDR includes those cases graders considered “active DMO”, “unsure” and “ungradable” based on 7-field ETDRS images; G-OPTOS referral for PDR = referral for PDR includes those cases graders considered “active DMO”, “unsure” and “ungradable” based on ultra-wide field Optos images.

ETDRS = Early Treatment Diabetic Retinopathy

Supplementary Table A10: Cost comparison of ophthalmic grader pathway and standard care for proliferative diabetic retinopathy (SENA 4)

Sensitivity Analysis (SENA) Scenario	Sensitivity	Specificity	Cost per 100 patients	Cost saving compared to standard care
**PDR with pre-retinal or vitreous haemorrhage in either eye based on the reference standard***	Assumed 100%	Assumed 100%	£5,800.00	-
**G-ETDRS referral for PDR**	80.00%	40.00%	£5,803.00	-£3.00
**G-OPTOS referral for PDR**	87.00%	49.00%	£4,843.00	£957.00

* = ophthalmologist face-to-face examination; G-ETDRS referral for PDR = referral for PDR includes those cases graders considered “active DMO”, “unsure” and “ungradable” based on 7-field ETDRS images. G-OPTOS referral for PDR = referral for PDR includes those cases graders considered “active DMO”, “unsure” and “ungradable”, based on ultra-wide field Optos images

ETDRS = Early Treatment Diabetic Retinopathy

Savings would be modest or even zero in SENA 4 (Table A10) for ophthalmic graders evaluating 7-field ETDRS images for people with pre-retinal or vitreous haemorrhages (i.e. high-risk PDR) requiring treatment. There would be savings with UWF imaging. In this group, sensitivity was 87% for UWF images (95% CI 78-93%) and 80% (CI 69-88%) for 7-field ETDRS images and specificities were 49% and 40%, respectively.

Supplementary Table A11: Cost comparison of ophthalmic grader pathway and standard care for proliferative diabetic retinopathy (Additional Post-hoc Analysis)

	Sensitivity	Specificity	Cost per 100 patients	Cost saving compared to standard care
**Active PDR in either eye based on the reference standard* + O-ETDRS**	Assumed 100%	Assumed 100%	£9,236.00	-
**Active PDR in either eye based on the reference standard* + O-OPTOS**	Assumed 100%	Assumed 100%	£8,598.00	
**G-ETDRS referral for PDR**	84.00%	52.00%	£5,107.00	£4,129.00
**G-OPTOS referral for PDR**	81.00%	57.00%	£4,379.00	£4,219.00

* = ophthalmologist face-to-face examination; O-ETDRS = ophthalmologist assessment of ETDRS 7-field images; O-Optos = ophthalmologists assessment of ultra-wide field Optos images; G-ETDRS referral for PDR = referral for PDR includes those cases graders considered “active DMO”, “unsure” and “ungradable” based on 7-field ETDRS images. G-OPTOS referral for PDR = referral for PDR includes those cases graders considered “active DMO”, “unsure” and “ungradable”, based on ultra-wide field Optos images.
